# Effects of Diabetes Mellitus on Motor and Non-Motor Symptoms in Parkinson’s Disease: A Cross-Sectional Study

**DOI:** 10.31083/RN38630

**Published:** 2025-08-27

**Authors:** Irving C. Rodríguez-González, Fernando Botello-Villagrana, Sergio R. Gomez-Villalobos, Ximena M. Torres-Mancilla, Arnulfo González-Cantú, Mirna González-González, Daniel Martinez-Ramirez

**Affiliations:** ^1^Tecnologico de Monterrey, School of Medicine and Health Sciences, 64710 Monterrey, Nuevo León, Mexico; ^2^Tecnologico de Monterrey, School of Medicine and Health Sciences, 44379 Guadalajara, Jalisco, Mexico; ^3^Christus Muguerza Alta Especialidad, 64780 Monterrey, Nuevo León, Mexico

**Keywords:** Parkinson’s disease, diabetes mellitus, motor symptoms, non-motor symptoms, cognitive dysfunction, enfermedad de Parkinson, diabetes mellitus, síntomas motores, síntomas no-motores, deterioro cognitivo

## Abstract

**Introduction:**

While there is a growing body of evidence indicating a potential connection between Parkinson’s disease and diabetes mellitus, there is a lack of focus on investigating how diabetes correlates with the severity of both motor and non-motor symptoms in Parkinson’s disease.

**Objective:**

This study examined and contrasted both motor and non-motor symptoms in patients diagnosed with Parkinson’s disease, stratified by the presence or absence of diabetes.

**Methods:**

A total of 40 Parkinson’s disease patients, divided into two groups (with and without diabetes), were assessed using various scales, including the Movement Disorders Society – Unified Parkinson’s Disease Rating Scale, Scales for Outcomes in Parkinson’s Disease - Autonomic Dysfunction and Non-Motor Symptoms, Beck Depression Inventory, Montreal Cognitive Assessment, and Parkinson’s Disease Questionnaire-39. Demographic and clinical characteristics were also recorded. Statistical analyses included *t*-tests, Mann-Whitney U tests, and Fisher’s exact test.

**Results:**

Significant differences were observed in the motor sub-score of postural instability and gait disturbance symptoms, autonomic total scores, urinary function domain, depression scores, and quality of life in the mobility and emotional domains between the Parkinson’s disease non-diabetes, and Parkinson’s disease - diabetes groups.

**Conclusions:**

Our study unveiled differences in motor and Non-Motor Symptoms among patients with Parkinson’s disease and diabetes, underscoring the influence of diabetes on manifestations of the disease.

## Abstract (es)

**Introducción:**

Existe creciente evidencia sobre la posible conexión entre el Parkinson y la Diabetes Mellitus. Todavía se requiere mayor investigación para comprender la relación entre la diabetes y la severidad de los síntomas en la enfermedad de Parkinson.

**Objetivo:**

Comparar los síntomas motores y no motores en pacientes con enfermedad de Parkinson con y sin diabetes.

**Métodos:**

Se evaluaron 40 pacientes con Parkinson, divididos en dos grupos (con y sin diabetes), utilizando varias escalas clínicas para evaluar sintomatología motora y no motora de la enfermedad. Se registraron características demográficas y clínicas. Se realizaron análisis estadísticos que incluyeron pruebas t, pruebas U de Mann-Whitney y la prueba exacta de Fisher.

**Resultados:**

Se observaron diferencias significativas en la sub-puntuación motora de los síntomas de inestabilidad postural y trastorno de la marcha, puntuaciones totales autonómicas, dominio de la función urinaria, puntuaciones de depresión y calidad de vida en los dominios de movilidad y emocional entre los grupos de enfermedad de Parkinson sin diabetes y con diabetes.

**Conclusiones:**

El estudio reveló diferencias en los síntomas motores y no motores entre pacientes con enfermedad de Parkinson y diabetes, resaltando la influencia de la diabetes en las manifestaciones de la enfermedad.

## 1. Introduction

Parkinson’s disease (PD) and diabetes mellitus (DM) are two common chronic conditions that play a major role in the growing burden of global health. In 2023, the worldwide prevalence of PD across all ages was estimated at 151 per 100,000 people—roughly 8.5 million individuals—making it the second most common neurodegenerative disease after Alzheimer’s disease, which affects around 6.9 million Americans aged 65 and older [1, 2]. In contrast, DM displayed a global prevalence of 475,995.8, with 462,976.9 cases attributed to type 2 diabetes mellitus (DM2) [3]. Patients with DM2 have been reported to have a higher risk of developing PD [4, 5, 6, 7, 8, 9, 10]. Common pathways between the two diseases, such as inflammatory pathways and oxidative stress, have been documented [11, 12]. In experimental models, insulin can modulate dopaminergic activity, and prolonged exposure to hyperglycemia induces dopaminergic alterations [13, 14]. As global PD rates rise, understanding its development and severity factors is crucial. While evidence suggests a PD-DM link, little attention focuses on DM’s impact on PD symptom severity, including motor and non-motor symptoms.

In Mexico, both PD and type 2 diabetes are emerging as major clinical concerns. Current national data suggest an incidence rate of approximately 37.9 cases per 100,000 people, predominantly among the elderly [15]. However, due to the absence of comprehensive, long-term population studies, there are still no official estimates of its overall prevalence. On the other hand, type 2 diabetes is notably widespread, affecting 18.3% of the adult population, a figure that includes many undiagnosed individuals [16]. The coexistence of both conditions in patients adds further complexity to disease progression, symptom management, and quality of life. This underscores the urgent need to understand how diabetes may influence the clinical presentation of PD.

In a case-control investigation involving 39 individuals diagnosed with PD, those who also had DM demonstrated more unfavorable outcomes in terms of bradykinesia (13.7 in the DM group compared to 12.3 in the non-DM group, with a *p *= 0.0023), rigidity (5.0 in the DM group compared to 4.6 in the non-DM group, with a *p *= 0.048), and postural instability and gait difficulties (5.0 in the DM group compared to 3.6 in the non-DM group, with a *p* ≤ 0.0001) [17]. Likewise, in another case-control research study that tracked PD patients over a three-year period, it was observed that individuals with DM displayed significant distinctions in both motor and non-motor symptoms as assessed by the Movement Disorder Society Unified Parkinson’s Disease Rating Scale (MDS-UPDRS) [18]. According to the authors, the presence of DM before the onset of PD seems to be associated with a higher risk of experiencing more severe PD symptoms. A recent comprehensive systematic review and meta-analysis suggested that there is a significant association between type 2 DM and the accelerated progression of motor symptoms in PD [19].

Three studies have examined the impact of DM on the cognition of PD patients. The first study found that PD patients with DM had more severe cognitive impairment than those without DM, with lower global cognition scores in the diabetes group [20]. A second study reported that PD patients with diabetes had a significantly smaller total gray matter volume and lower scores in visuospatial, executive, and composite domains [21]. The last study observed significant cognitive changes over 36 months in both PD patients with and without diabetes, with substantial declines in mini-mental state examination (MMSE) and Montreal Cognitive Assessment (MoCA) scores in both groups [22]. In summary, these studies indicate a potential detrimental effect of DM on the cognitive abilities of individuals with PD, potentially resulting in more severe cognitive impairment and structural brain alterations. However, it is important to note that a systematic review and meta-analysis did not identify substantial evidence of accelerated cognitive decline in PD patients with DM [19]. This highlights a literature gap, emphasizing the need for more research to confirm diabetes’ impact on cognitive impairment in PD patients.

This study aims to assess DM’s effect on PD symptom severity, exploring both motor and non-motor symptoms. Results not only deepen our understanding of DM’s impact on PD symptoms but also have practical and societal implications. They may guide future research on therapeutic interventions.

## 2. Subjects and Methods

We conducted an observational cross-sectional study at the movement disorders clinic overseen by the principal investigator, spanning from January to June 2022. Ethical approval was secured from the institutional ethics committee (No. P000415-DMPARK-CEIC-CR003). Participants were adults aged 18 or older with a confirmed diagnosis of PD made by a neurologist specializing in movement disorders. All individuals gave informed consent and demonstrated proficiency in the Spanish language. Exclusion criteria included the presence of severe psychiatric symptoms, refusal to provide consent, or major neurocognitive disorders, as defined by diagnostic and statistical manual of mental disorders, fifth edition (DSM-5) guidelines—marked cognitive impairments in areas like memory, attention, language, or executive functioning that significantly hinder daily independence. This was essential to ensure valid responses on self-administered tools such as the MoCA, Beck Depression Inventory-II (BDI-II), and Parkinson’s Disease Questionnaire (PDQ-39), and to verify the ability to provide informed consent. Importantly, individuals with mild cognitive impairment were not excluded, as one of the central aims of the study was to evaluate non-motor symptoms, including cognitive decline.

The diagnosis of diabetes mellitus was confirmed using clinical records, following the criteria set by the American Diabetes Association (ADA), which include fasting plasma glucose levels ≥126 mg/dL, HbA1c values ≥6.5%, or the documented use of antidiabetic medications. This study exclusively included patients diagnosed with DM2, based on clinical histories and prescribed treatments [23]. The onset of diabetes was determined through a review of both patient medical records and electronic health data. Individuals with DM1 were excluded, due to their distinct pathophysiological characteristics and lower incidence in the target age group. This exclusion aimed to maintain sample uniformity and minimize confounding factors related to differing disease mechanisms and treatment regimens.

Data collected included somatometric measurements such as weight, height, body mass index (BMI), and waist circumference, sociodemographic information, patient history, clinical data and DM2 treatment details, if the case. Clinical characteristics of PD were also documented. The MDS-UPDRS was applied [24], along with various scales and questionnaires assessing motor and non-motor symptoms, including the Scales for Outcomes in Parkinson’s Disease-Autonomic Dysfunction (SCOPA-AUT) [25], Non-Motor Symptoms Scale for Parkinson’s Disease (NMSS) [26], BDI-II [27], MoCA [28], and PDQ-39 [29]. Clinical motor sub-scores were calculated from the MDS-UPDRS part III, including items for bradykinesia, tremor, rigidity, and postural instability and gait. Patients were categorized as obese if their BMI was ≥30, and those with a BDI-II score >17 points were classified as having depressive disorder. MoCA scores <26 points were indicative of cognitive impairment. Independence in daily life was assessed with a Schwab and England Scale percentage ≥80% [30].

### Statistical Analysis

Descriptive statistics were used to summarize means, standard deviations, medians, and interquartile ranges, as appropriate. The Shapiro–Wilk test assessed normality of continuous variables. Categorical variables were analyzed using either the chi-square or Fisher’s exact test, while comparisons of continuous variables between PD-DM and PD non-DM groups employed Student’s *t*-test or the Mann–Whitney U test, depending on data distribution. Statistical significance was defined as *p* < 0.05. Analyses were performed using IBM SPSS Statistics version 25 (IBM-SPSS Statistics, Chicago, IL, USA).

## 3. Results

A total of 40 PD patients were included in the study, with 20 having DM and 20 without. Table 1 shows sociodemographic and clinical characteristics of the study cohort. Among them, 57.5% (n = 23/40) were male, and the mean age was 66.6 ± 8.96 years. Regarding occupation, 37.5% (n = 15/40) were homemakers, 32.5% were employed (n = 12/40), and 30% were retired or pensioned (n = 13/40). The mean weight was 72.8 ± 11.4 kg, with an average waist circumference of 83.5 ± 10.4 cm. Furthermore, 32.5% (n = 13/40) were overweight, and 30% (n = 12/40) were classified as obese (BMI ≥30). Clinical characteristics of the PD patients revealed an average disease duration of 65.1 ± 37 months. The most used medication for PD treatment was levodopa associated with a dopa decarboxylase inhibitor in 92.5% of cases, followed by dopamine agonists in 60%, amantadine in 15%, type-B monoamine oxidase (MAOB) inhibitors in 12.5%, and catechol-O-methyltransferase (COMT) inhibitors in 5%. The mean daily levodopa dose was 916.1 ± 343 mg. In terms of disease motor subtype, 40% exhibited postural instability and gait disturbance (n = 16/40), 35% displayed a tremor-predominant subtype (n = 14/40), and 25% were categorized as indeterminate (n = 10/40). Regarding the etiology of PD, 72.5% were considered idiopathic (n = 29/40), while 27.5% (n = 11/40) had a positive family history of PD. As for disease stage according to the Hoen and Yahr scale, 27.5% were in stage 3 (n = 11/40), 32.5% were in stage 2 (n = 13/40), and 27.5% in stage 1 (n = 11/40). The mean score for part III of the MDS-UPDRS in the on-medication state was 28.1 ± 17.6 points. The cohort reported motor fluctuations in 50% of patients (n = 20/40) and dyskinesias in 27.5% (n = 11/40). Regarding non-motor symptoms, in part I of the MDS-UPDRS, the mean score was 15.2 ± 8.6 points, and in part II, it was 12.2 ± 11.2 points. All patients presented at least one non-motor symptom. The NMS scale had a mean score of 57.5 ± 41.3 points, and SCOPA-AUT had a mean of 24.9 ± 11.4 points. The mean MoCA score for cognitive assessment was 24.5 ± 4.1 points, primarily indicating executive and visuospatial impairment. The BDI-II showed a mean score of 12.9 ± 8.7 points. According to the Schwab & England scale, 70% had a percentage ≥80% for independence in daily life. In the PDQ-39, the mean score across the eight domains was 4.9 ± 3.2 points. For patients with DM, the mean duration of diabetes was 106.8 ± 73.1 months. Medications used included metformin in 42.5% (n = 17/40), dipeptidyl peptidase IV (DPP4) inhibitors in 17.5% (n = 7/40), insulin in 7.5% (n = 3/40), sulfonylureas in 5% (n = 2/40), and sodium-glucose cotransporter-2 (SGLT2) inhibitors in 2.5% (n = 1/40).

**Table 1.** **Demographic and general clinical characteristics of patients with PD non-DM and PD-DM**.

	PD non-DM (n = 20)	PD-DM (n = 20)	*p*-value
Age, years, mean (SD)*	64.1 (10.1)	69.1 (7.1)	0.080
Male, n (%)	9 (45)	14 (70)	0.257
H&Y, median (IQR)+	1.95 (1.2)	2.2 (0.9)	0.550
Disease duration, months, median (IQR)+	60 (66)	42 (30)	0.760
Levodopa dailly dose, mg, median (IQR)+	850 (487.5)	1000 (562.5)	0.230
Weight, kg, mean (SD)*	70.5 (11.7)	75.9 (10.4)	0.120
Abdominal perimeter, cm, mean (SD)*	80.5 (8.7)	86.4 (11.4)	0.070
Obesity, n (%)++	3 (15)	5 (25)	0.460

*Independent *t*-test, +Mann-Whitney U test, ++ Fisher’s Exact Test. PD, Parkinson’s disease; DM, diabetes mellitus; SD, standard deviation; H&Y, Hoehn & Yahr; IQR, Interquartile Range.

Comparison between PD patients with and without DM did not reveal significant differences in terms of age, sex, Hoehn and Yahr stage, disease duration, daily levodopa dosage, weight, waist circumference, or the presence of obesity. In the motor domain, there were no significant differences in MDS-UPDRS part III scores, except for a significant difference observed in the postural instability and gait disturbance sub-score (*p *= 0.043). Part IV of the MDS-UPDRS, presence of dyskinesias, and motor fluctuations did not show significant differences. Non-motor symptom assessments, including MDS-UPDRS part I and MoCA, did not yield significant differences between the two groups.

However, a significant difference was noted in the total SCOPA-AUT score, where those without DM reported a median of 17 (22.5) compared to 25.5 (11.5) for those with DM, with a *p* = 0.026. Furthermore, the NMS scale demonstrated a significant difference in the urinary function domain, with a median of 3 (6) for those without DM versus 8.5 (7) for those with DM, with a *p *= 0.003. In the BDI-II, a significant difference was observed between the two groups, with a median of 9 (11) for those without DM and 11 (16.5) for those with DM, with a *p *= 0.012. While there was no significant difference in the overall PDQ-39 score, sub-scores indicated a significant difference in the mobility domain, with those with DM reporting a higher score for mobility issues (moving at home and in public places), with a median of 5 (19) compared to 14 (5) for those without DM, with a *p *= 0.048. Additionally, a higher score in the emotional domain (mood) was reported by those with DM, with a median of 4 (9) compared to 9 (10.5) for those without DM, with a *p* = 0.01 (Table 2).

**Table 2.** **Motor, non-motor, and quality of life characteristics of patients with PD non-DM and PD-DM**.

	PD non-DM (n = 20)	PD-DM (n = 20)	*p*-value
MDS-UPDRS part II, median (IQR)+	7.5 (19)	9.5 (13)	0.720
MDS-UPDRS part III, median (IQR)+	18.5 (27.5)	26 (13)	0.220
Tremor sub-score, median (IQR)+	5.5 (6)	5 (4.5)	0.880
Rigidity sub-score, median (IQR)+	3.5 (4)	4 (3)	0.210
Bradykinesia, median (IQR)+	10 (11.5)	10 (5)	0.580
Postural instability and gait difficulty sub-score, median (IQR)+	1 (7)	5.5 (3)	0.043
MDS-UPDRS part IV, median (IQR)+	0 (11)	1.5 (10)	0.620
Total, MDS-UPDRS, median (IQR)+	49.5 (75.5)	56.5 (38.5)	0.410
Dyskinesias, n (%)++	6 (30)	5 (25)	1.000
Motor/non-motor fluctuations, n (%) ++	9 (45)	10 (50)	1.000
MDS-UPDRS part I, mean (SD)*	13.6 (9.3)	16.8 (7.7)	0.120
SCOPA-AUT total score, median (IQR)+	17 (22.5)	25.5 (11.5)	0.026
NMS scale total score, median (IQR)+	34.5 (65.5)	55 (55.5)	0.085
Cardiovascular/falls domain, median (IQR)+	1 (2.5)	1 (3.5)	0.400
Sleep/fatigue domain, median (IQR)+	4 (7.5)	6.5 (9)	0.450
Mood/cognition domain, median (IQR)+	2 (15.5)	5 (13.5)	0.320
Perceptual problems/hallucinations domain, median (IQR)+	0	0	1.000
Attention/memory domain, median (IQR)+	0 (9)	3 (6.5)	0.580
Gastrointestinal tract domain, median (IQR)+	4 (5)	4.5 (4)	0.130
Urinary domain, median (IQR)+	3 (6)	8.5 (7)	0.003
Sexual function domain, median (IQR)+	11.5 (11)	18 (10)	0.130
Miscellaneous domain, median (IQR)+	4 (9)	5.5 (4.5)	0.240
BDI-II, median (IQR)+	9 (11)	11 (16.5)	0.012
MoCA total score, median (IQR)+	26 (7)	23 (4.5)	0.130
MoCA, <26, n (%) ++	9 (45)	13 (65)	0.340
PDQ-39 total score, median (IQR)+	1.5 (6.6)	5.2 (3.7)	0.080
Mobility domain, median (IQR)+	5 (19)	14 (5)	0.048
Activities of daily living domain, median (IQR)+	3 (11)	6.5 (2.5)	0.190
Emotional well-being domain, median (IQR)+	4 (9)	9 (10.5)	0.010
Stigma domain, median (IQR)+	0	0	1.000
Social support domain, median (IQR)+	0	0	1.000
Cognitions domain, median (IQR)+	1.5 (7)	4.5 (5)	0.150
Communication domain, median (IQR)+	0.5 (3)	2 (4)	0.330
Bodily discomfort domain, median (IQR)+	4 (4)	4.5 (4.5)	0.540
Schwab & England Scale <80%, n (%)++	5 (25)	7 (35)	0.730

*Independent *t*-test, +Mann-Whitney U test, ++ Fisher’s Exact Test. MDS-UPDRS, Movement Disorder Society Uni- fied Parkinson’s Disease Rating Scale; SCOPA-AUT, Scales for Outcomes in Parkinson’s Disease–Autonomic; NMS, Non-Motor Symptoms Scale; BDI-II, Beck Depression Inventory-II; MoCA, Montreal Cognitive Assessment; PDQ-39, Parkinson’s Disease Questionnaire.

## 4. Discussion

In this observational cross-sectional study, we compared the motor and non-motor symptoms of PD between patients with and without DM. We observed significant differences in the following aspects: (1) the motor sub-score related to symptoms of postural instability and gait disturbance as assessed by the MDS-UPDRS III, (2) the autonomic function scores, encompassing total SCOPA-AUT scores and the urinary function domain within the NMS scale, (3) Beck Depression Inventory scores, and (4) the domains of mobility and emotional well-being when evaluating quality of life. Our results indicated that PD patients with DM tended to experience more severe symptoms in these areas.

An additional mechanism that may warrant further consideration is Diabetic Striatopathy (DS)—a condition that has gained recognition as a hyperglycemia-associated movement disorder, characterized by striatal dysfunction and distinct T1-weighted MRI alterations. While DS was historically linked to chorea-ballism, a notable clinical series by Dubey* et al*. (2022) [31] expanded its spectrum to include other movement disorders, including parkinsonism, in individuals with poorly controlled diabetes [32]. DS is believed to stem from glucose-driven metabolic disturbances in the basal ganglia, especially the putamen and caudate nuclei, leading to gamma-aminobutyric acid (GABA) depletion and compromised thalamocortical inhibition. Notably, nearly one-third of patients in that study presented with non-choreic manifestations, such as parkinsonism—findings particularly relevant to our cohort. This supports the hypothesis that persistent or subclinical striatal damage due to chronic hyperglycemia may aggravate dopaminergic deficits, potentially intensifying motor symptoms like postural instability and gait disturbance. Such overlap suggests a converging pathophysiology between DS and Parkinsonian features, especially in patients with inadequate glycemic control.

Our findings revealed that the presence of DM did not significantly impact the motor symptoms of PD when assessed by the MDS-UPDRS III total score. However, when we examined motor sub-scores of the MDS-UPDRS III, we observed that patients with DM had higher scores in items related to postural instability and gait disturbance. This observation is consistent with a previous study [17], which described greater impairment in postural instability and gait disturbance scores among PD patients with DM. The observed link between DM and the heightened motor symptoms associated with postural instability and gait disturbance in PD patients can be attributed to several underlying factors. One plausible explanation involves the presence of vascular pathology in both DM and PD. These conditions share common risk factors such as hypertension, hyperlipidemia, and insulin resistance, which can culminate in vascular dysfunction and microvascular damage [33]. Additionally, DM’s impact on peripheral neuropathy is another potential contributor. DM can induce damage to sensory and motor nerves, impairing proprioception and muscle control [34]. Finally, it is conjectured that this association may be influenced by other mechanisms of neuronal damage independent of the nigrostriatal dopaminergic pathway.

Regarding non-motor symptoms, our study indicated that PD patients with DM had higher scores on the dysautonomia scale, primarily in the urinary function domain, including urinary incontinence and nocturia. No significant differences were observed in other domains of dysautonomia. Similarly, a recent study reported significant differences in the NMSS score between PD diabetics and non-diabetics [35]. However, these findings contradict a previous study [36], which evaluated patients with controlled and uncontrolled DM alongside PD and found no significant differences in scores. The observation of increased dysautonomia, particularly in the urinary function domain, among PD patients with DM in our study can be elucidated through several theoretical mechanisms. One potential explanation relates to the common pathophysiological factors shared between DM and dysautonomia in PD. Both conditions have been associated with autonomic dysfunction and alterations in sympathetic and parasympathetic nervous system regulation [37]. Furthermore, DM-related microvascular and macrovascular complications, such as neuropathy and vascular dysfunction, could contribute to urinary symptoms in PD patients with DM [38].

Our findings revealed a notably higher impact on Beck’s Depression Inventory score among patients with PD-DM. This observation aligns with previous reports indicating that individuals living with both PD and DM tend to experience a more pronounced impact of depression [39]. One plausible explanation is the potential connection between defective brain insulin signaling and depression. Underlying molecular mechanisms include impairments in the reward system, neurogenesis, synaptic plasticity, and regulation via the hypothalamic-pituitary-adrenal (HPA) axis [40, 41]. Additionally, tumor necrosis factor alpha (TNF-α) has been implicated in disrupting insulin signaling and promoting depressive-like behaviors in preclinical models [42].

Given these underlying pathophysiological links, recent clinical trials have started to investigate whether antidiabetic medications might have disease-modifying properties in PD. Notably, the phase 2 randomized LIXIPARK trial assessed lixisenatide—a GLP-1 receptor agonist typically prescribed for type 2 diabetes—as a possible disease-modifying intervention in early-stage PD. Although individuals with diabetes were excluded, the study found that lixisenatide significantly reduced motor disability progression over 12 months compared to placebo. Specifically, the mean change in MDS-UPDRS part III scores in the on-medication condition was –0.04 for the lixisenatide group versus +3.04 for placebo, yielding a statistically significant difference of 3.08 points (*p *= 0.007). Furthermore, after a two-month washout, motor scores in the off-medication state remained lower in the treatment group (17.7 vs. 20.6), further pointing to a possible disease-modifying effect. However, no notable changes were observed in non-motor symptoms or quality of life, and gastrointestinal side effects were frequent among those receiving active treatment. While the present study did not examine GLP-1–based therapies, these results reinforce the idea that metabolic mechanisms—particularly insulin-related pathways—could play a role in shaping PD progression and symptomatology (Meissner *et al*., 2024) [43].

The psychosocial burden of living with two chronic conditions, PD and DM, may also play a key role in the development of depressive symptoms [44]. The complexity of managing both diseases—medications, dietary restrictions, and complications—can result in elevated stress and reduced quality of life, which in turn predispose patients to depression. Moreover, the presence of physical disability and limited mobility due to PD may intensify feelings of helplessness, further contributing to the elevated BDI-II scores observed in this subgroup [45].

Beyond mood-related symptoms, growing research points to a potential role of diabetes mellitus in speeding up cognitive decline among people with PD. Persistent high blood sugar and insulin resistance can cause damage to small blood vessels in the brain, disrupting both cortical and subcortical networks essential for memory and executive functions. Additionally, the metabolic strain linked to diabetes may worsen mitochondrial performance, heighten oxidative stress, and promote low-level inflammation—all factors already tied to the neurodegeneration seen in PD. These intersecting processes might help explain why cognitive deterioration can progress more rapidly in patients living with both conditions. To better understand this connection, future studies using brain imaging and detailed cognitive assessments will be essential.

While our study provides valuable insights into the relationship between DM and PD, certain limitations must be acknowledged before interpreting the results. First, the study’s sample size was relatively small and drawn from a specific segment of the population, potentially limiting the generalizability of our findings to a broader demographic. Finally, it’s important to note that, given the modest sample size of this study, *p*-values hovering near the conventional cutoff for significance (e.g., *p *= 0.04–0.05) should be interpreted with some caution. The results may reflect random variation, and validation in larger, well-powered cohorts will be essential to confirm these associations. Additionally, due to the observational nature of our study, we were unable to establish a causal relationship between DM and the observed differences in motor and non-motor symptoms. Moreover, most diabetic patients in our sample were treated with metformin, preventing us from exploring the potential impact of different antidiabetic medications on PD symptoms. Another relevant limitation relates to pharmacological treatment differences between groups. Although overall patterns of dopaminergic medication use were noted, the study did not include direct comparisons between PD patients with and without diabetes in terms of specific drug types or usage frequency. This oversight is a significant limitation, as variations in treatment such as elevated levodopa dosages or the addition of adjunctive therapies may shape both motor and non-motor symptom profiles. Addressing treatment heterogeneity in future research will be essential to disentangle the specific impact of diabetes on Parkinsonian symptoms. Nonetheless, our study possesses notable strengths, including a comprehensive assessment of non-motor symptoms and quality of life, providing additional perspectives beyond the existing literature on this unique profile of patients with both PD and DM.

## 5. Conclusions

Our findings suggest that DM may impact PD regarding postural instability, gait disturbance symptoms, and the presence and severity of autonomic dysfunctions and depressive symptoms. Emphasizing preventive measures to reduce fall risks in PD patients with DM is recommended. Screening for depression in these patients is crucial. Prospective studies are needed to confirm these findings and better understand the interaction between DM and PD.

## Availability of Data and Materials

The dataset used and analyzed during the current study is part of a proprietary database and is not publicly available. However, it is available from the corresponding author upon reasonable request.
